# Essential oil composition, morphological characterization, phenolic content and antioxidant activity of Iranian populations of *Hymenocrater longiflorus* Benth. (Lamiaceae)

**DOI:** 10.1038/s41598-024-57826-0

**Published:** 2024-03-27

**Authors:** Basireh Fattahpour, Mohammad Fattahi, Abbas Hassani

**Affiliations:** Department of Horticulture, Faculty of Agriculture, Urmia University, Urmia, Iran

**Keywords:** Hierarchical cluster analysis, Canonical correspondence analysis, DPPHsc IC50, Total anthocyanin content, Total phenolic contents, Total flavonoid contents, Essential oil, Mono (2-ethylhexyl) phthalate, Secondary metabolism, Plant ecology, Natural variation in plants, Natural products, Screening

## Abstract

The study focused on the morphological and chemical characteristics of 200 *Hymenocrater longiflorus* Benth. genotypes found in natural habitats of eight regions in west of Iran. The primary objective of the study was to assess the morphological and phytochemical variability within populations grown in their natural habitats, with the aim of identifying their potential for domestication and utilization in pre-breeding programs. The plant height (PH) ranged from 50.32 to 69.65 cm, with the highest observed in population P8. The internode distances ranged from 4.7 to 6.47 cm, with the maximum distance found in P4. Flower lengths varied from 1.95 to 2.45 cm, with the minimum and maximum values observed in P4 and P3, respectively. The highest leaf length (5.20 cm) and width (3.87 cm) were recorded in P2. The aerial parts of the plant were utilized to extraction and determine the essential oil (EO) content and composition, which ranged from 0.40 to 0.78% (v/w). The analysis of EO by gas chromatography (GC) and gas chromatography mass spectrometry (GC/MS) identified 26 compounds, constituting 99–99.5% of the EOs. The main compounds in the EO and their percentage range (v/w DW) were tau-cadinol (0.62–55.56), mono (2-ethylhexyl) phthalate (8.10–94.70), elemol (0.21–19.11), β-spathulenol (0.08–14.39), 4-terpineol (0.23–10.19), and β-eudesmol (0.21–9.94). The main chemical groups found in EOs included oxygenated sesquiterpenes (1.12–68.43), and phthalates (9.73–94.72). Cluster analysis revealed three distinct chemotypes: chemotype I (populations 1 and 2) with major components of mono (2-ethylhexyl) phthalate, tau-cadinol, and α-elemol; chemotype II (population 5) rich in mono (2-ethylhexyl) phthalate; and chemotype III (populations 3, 4, 6–8) containing tau-cadinol, β-eudesmol, and 4-terpineol. The study also evaluated total phenolic, total flavonoid, and DPPH free radical scavenging activity in the fifty percent inhibitory concentration (IC50) in leaf and flower samples of the genotypes, along with estimating total anthocyanin content in the flower samples. The total phenolic content (TPC) in leaf and flower samples ranged from 7.89 to 107.18 mg GAE/g DW and 39.98 to 86.62 mg gallic acid equivalent (GAE)/g DW, respectively. Total flavonoid content (TFC) ranged from 81.04 to 143.46 mg QUE/g DW in leaf samples and from 94.82 to 133.26 mg quercetin equivalent (QUE)/g DW in flower samples. DPPHsc IC50 (µg/mL) ranged from 0.65 to 78.74 in leaf samples and from 4.38 to 7.71 in flower samples. Anthocyanin content ranged from 1.89 to 3.75 mg cyanidin-3-glucoside equivalent (C3GE)/g DW among populations. Canonical correspondence analysis and simple correlation demonstrated a strong association and correlations among the studied attributes. The negative correlations between leaf DPPH (DPPH L) IC50 and TFC (− 0.73), TPC (− 0.63), Elemol (− 0.90), and EO (− 0.85) indicate that these compounds have a significant impact on the antioxidant activity of the leaves. Furthermore, Fruit DPPH (DPPH F) IC50 showed a negative correlation with TPC (− 0.79) and TFC (− 0.78), but a positive correlation with flower anthocyanins (0.51), (Z)-β-Farnesene (0.66), and 4-Terpineol (0.57). Circular cluster analysis categorized the genotypes of all individuals in the eight studied populations into three main categories based on all the studied traits, indicating significant variation in phytochemical and morphological traits among populations, surpassing the within-populations variation.

## Introduction

Plants have significantly contributed to the longevity of human life by serving as medicinal sources for the treatment and prevention of various diseases. In recent years, there has been a notable surge in the utilization of plants and their derivatives within the food industry and for nutraceutical applications^1–4^. The utilization of plants as cooked products or raw materials, either fresh or dried, as whole plants or in parts, has been a common practice. The Lamiaceae family plays a crucial role within the plant kingdom due to its significant importance as a source of medicinal, aromatic, spice, and edible plants^5^. This family comprises more than 220 genera across 4,000 species, many of which possess medicinal properties and contain essential oils^6^. Essential oils, which are concentrated liquids containing volatile chemical components with aromatic properties, are extracted from different parts of plants as secondary metabolites. These components possess essential ecological and biological properties. The quality and quantity of essential oil can be influenced by various factors, such as genetics, nutrition, sunlight, temperature, humidity, location, and harvesting time^7,8^. The Lamiaceae family includes the genus *Hymenocrater*, which consists of more than 21 species found worldwide. Within Iran, there are nine naturally occurring species, with five of them being endemic to the country. These species are *H. incanus* Bunge, *H. oxyodontus* Rech. F., *H. yazdianus* Rech. F., *H. platystegius* Rech. F., and *H. calycinus* (Boiss.) Benth^9^. One particular species, *H. longiflorus*, is endemic to the western regions of Iran and eastern Iraq^10–12^. It thrives at altitudes ranging from 1550 to 2300 m above sea level in the Iranian plateau. In Iran, this particular plant is commonly referred to as Oraman's tulip or Gole Arvaneh-Avarmani. However, in the western part of the country, locals often refer to it as Soor-Halale or surehahala^11–13^. *H. longiflorus* is a densely packed, highly fragrant plant with green leaves and purple or light purple flowers. In the Zagros region, locals utilize the aerial parts of this plant in raw or baked form for their anti-inflammatory properties, sedative effects, and to alleviate allergic skin reactions^12^. Both traditional medicine and recent pharmacological studies have recognized the plant as having a wide range of beneficial properties. These include antimicrobial, antifungal, antioxidant, cytotoxic, larvicidal, and mosquito repellent properties as well as home freshening abilities^10,12,14,15^. Previous studies have primarily focused on analyzing the chemical composition of this plant using a population collected exclusively from Paveh, Kermanshah with predominant compounds hedycaryol (22.2%), α-cadinol (20.43%), and β-bourbonnene (5.76%)^16^. Additionally, plants collected from Kurdistan-Iran, were found to contain α-pinene, β-caryophyllene, and β-ocimene, with percentages of 22.47, 18.05, and 14.92, respectively^12^. Another study conducted in Bayangan-Kermanshah reported the presence of **γ**-cadinol, α-pinene, and *p*-menth-1-en-8-ol, with percentages of 18.49, 10.16, and 9.82, respectively^15^. Furthermore, a specific investigation examined the expression patterns of specific genes involved in biosynthesis, including TPS 27, L3H, TPS2, TPS1, OMT, and GDH3, which are associated with the synthesis of 1,8-cineole, carvone, α-pinene, thymol, estragole, and β-citronellol. This study analyzed the gene expression under in vitro in drought stress conditions^13^. These findings contribute valuable insights into the chemical composition and genetic makeup of this plant. Despite the medicinal properties associated with *H. longiflorus*, there has been a lack of evidence regarding its morphological and phytochemical variations in Iran. Therefore, this study aims to investigate the morphological and phytochemical variations, as well as the essential oil composition, of 200 individuals of *H. longiflorus* plants grown in eight natural habitats. The study also intends to conduct multivariate analysis to further explore these variations and introduce of possible new chemotypes. A fundamental understanding of chemical compounds, as well as the morphological and antioxidant status of plants, is essential for identifying superior plant species for domestication and breeding programs.

## Results and discussion

**Figure 1:**
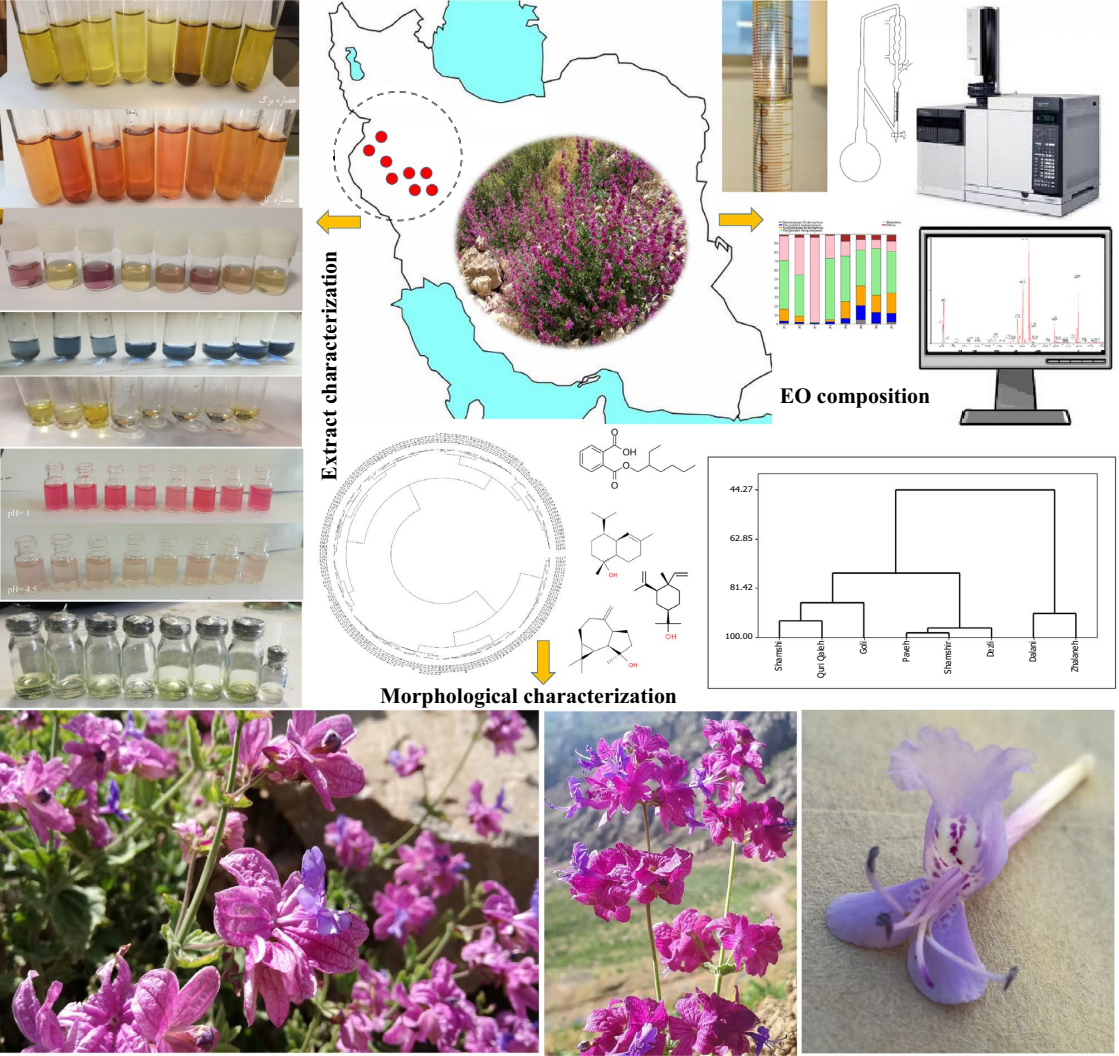
The different segments of plant and research stages design in the present study.

Considering that this plant is endemic to western parts of Iran and eastern regions of Iraq and no studies have been conducted on the populations of the plant and their genotypes, and on the other hand, due to the reported effects of antimicrobial, antifungal, antioxidant, cytotoxic, larvicidal, and mosquito repellent properties as well as home freshening abilities in this plant, we need to conduct further studies in this regard^8,10,12,13^. In this study, the chemical and physical diversity among 200 individuals from 8 populations of *H. longiflorus* plants was evaluated. Collection areas, genotypes numbers, geographical and topographical characteristics of *H. longiflorus* of 8 populations are shown in Table 1. In addition the design of this study is illustrated in Fig. 1.

**Table 1:** Collection areas, genotypes numbers, geographical and topographical characteristics of *H. longiflorus* populations.

Code	Genotypes	Provience	Populations	Longitude (E)	Latitude(N)	Altitude(M)	Habitat slope
P1	G 1–25	Kermanshah	Shamshi	46° 13´	35° 11´	2125	Eastern
P2	G 26–50	Kermanshah	Dalani	46° 12´	35° 08´	2326	Northern
P3	G 51–75	Kermanshah	Quri Qaleh	46° 31´	34° 54´	2335	Southern
P4	G 76–100	Kurdistan	Goli	46° 17´	35° 09´	1968	Northern-East
P5	G 101–125	Kurdistan	Zhalaneh	46° 11´	35° 10´	2157	Northern
P6	G 126–150	Kermanshah	Paveh	46° 22´	35° 10´	1940	Northern-West
P7	G 151–175	Kermanshah	Shamshir	24° 26´	35° 00´	2172	Western
P8	G 176–200	Kurdistan	Dezli	46° 09´	35° 15´	1775	Western

## Morphological dimension analysis

The average morphological characteristics of each population along with their standard error are shown in Table 2. The plant height (PH) ranged from 50.32 to 69.65 cm, with the highest observed in population P8. The number of nodes per stem (NNS) and the number of flowers per stem (FNS) were highest in population P8, with values of 16.40 and 57.92, respectively. The internode distances ranged from 4.7 to 6.47 cm, with the maximum distance found in P4. Flower lengths varied from 1.95 to 2.45 cm, with the minimum and maximum values observed in P4 and P3, respectively. The highest leaf length (5.20 cm) and leaf width (3.87 cm) were recorded in P2. Additionally, other measurements fell within the following ranges: Bract length (0.06 to 0.21 cm), Bract width (0.09 to 0.19 cm), Peduncle length (2.79 to 3.89 cm), Upper lip length (0.65 to 1.00 cm), and Calyx length (1.58 to 1.86 cm). The wide data ranges for all traits indicate significant diversity among the populations. The plant in question is currently classified as an endemic species that thrives under natural conditions. Given its significant medicinal value, it is crucial to cultivate and breed the plant to meet the growing demand. To achieve this goal, it is imperative to have a diverse range of plants, which facilitates the selection of suitable candidates for breeding purposes. Consequently, the existing diversity of the plant can be instrumental in this regard, as it provides a rich pool of genetic resources to draw from^17^.

**Table 2:** Morphological characteristics (Means ± standard errors) of *Hymenocrater longiflorus* of 200 genotypes of eight studied populations.

Morphological traits	Unit	P1 (G1-25)	P2 (G26-50)	P3 (G51-75)	P4 (G76-100)	P5 (G101-125)	P6 (G126-150)	P7 (G151-175)	P8 (G176-200)
PH: Plant height	cm	55.15 ± 1.56	56.76 ± 2.55	68.50 ± 2.42	50.32 ± 2.10	55.81 ± 2.20	62.94 ± 2.72	52.58 ± 1.62	69.65 ± 2.75
NNS: Node number per stem	–	14.48 ± 0.34	13.50 ± 0.36	15.36 ± 0.55	12.92 ± 0.33	15.75 ± 0.44	14.56 ± 0.47	15.20 ± 0.35	16.40 ± 0.51
FNS: Flower number per stem	–	34.40 ± 0.26	37.63 ± 2.40	54.20 ± 3.64	31.24 ± 2.00	32.08 ± 2.88	42.84 ± 4.16	45.96 ± 3.53	57.92 ± 4.21
ID: Internode distance	cm	5.65 ± 0.24	6.53 ± 0.28	5.86 ± 0.31	6.47 ± 0.22	4.85 ± 0.22	6.42 ± 0.26	4.70 ± 0.24	6.24 ± 0.27
BL: Bract length	mm	4.80 ± 0.21	4.52 ± 0.19	4.55 ± 0.15	4.05 ± 0.13	3.22 ± 0.14	3.59 ± 0.15	3.23 ± 0.06	3.22 ± 0.13
BW: Bract width	mm	4.01 ± 0.17	3.93 ± 0.19	3.49 ± 0.13	3.28 ± 0.10	2.48 ± 0.11	3.06 ± 0.12	2.44 ± 0.09	2.51 ± 0.13
SD: Stem diameter	cm	0.24 ± 0.01	0.28 ± 0.02	0.28 ± 0.02	0.24 ± 0.01	0.20 ± 0.00	0.24 ± 0.01	0.24 ± 0.02	0.21 ± 0.01
PL: Peduncle length	cm	3.89 ± 0.19	3.54 ± 0.19	3.70 ± 0.24	3.30 ± 0.22	2.83 ± 0.24	2.98 ± 0.23	2.79 ± 0.11	3.67 ± 0.24
FL: Flower length	cm	2.25 ± 0.05	2.32 ± 0.05	2.45 ± 0.04	1.95 ± 0.07	1.98 ± 0.03	2.28 ± 0.04	2.25 ± 0.03	2.39 ± 0.04
ULL: Upper lip length	cm	0.85 ± 0.03	0.91 ± 0.03	1.00 ± 0.03	0.75 ± 0.04	0.65 ± 0.04	0.80 ± 0.03	0.87 ± 0.05	0.88 ± 0.02
LLL: Lower lip length	cm	0.50 ± 0.02	0.59 ± 0.02	0.59 ± 0.02	0.46 ± 0.02	0.39 ± 0.02	0.46 ± 0.02	0.47 ± 0.02	0.48 ± 0.01
CalL: Calix length	cm	1.73 ± 0.07	1.75 ± 0.04	1.96 ± 0.03	1.58 ± 0.06	1.63 ± 0.03	1.87 ± 0.03	1.84 ± 0.02	1.94 ± 0.03
LL: Leaf length	cm	4.20 ± 0.10	5.20 ± 0.28	4.70 ± 0.07	4.53 ± 0.02	3.83 ± 0.11	3.97 ± 0.06	3.73 ± 0.02	3.70 ± 0.09
LW: Leaf width	cm	3.47 ± 0.12	3.87 ± 0.13	3.03 ± 0.04	3.50 ± 0.09	3.07 ± 0.11	3.13 ± 0.04	2.77 ± 0.03	2.77 ± 0.02
L:W: Length to width ratio	–	1.23 ± 0.02	1.31 ± 0.03	1.55 ± 0.01	1.33 ± 0.04	1.29 ± 0.05	1.25 ± 0.01	1.23 ± 0.04	1.20 ± 0.07

P1: Kermanshah, Shamshi; P2: Kermanshah, Dalani; P3: Kermanshah, Quri Qaleh; P4: Kurdistan, Goli; P5: Kurdistan, Zhalaneh; P6: Kermanshah, Paveh; P7: Kermanshah, Shamshir; P8, Kurdistan, Dezli.

### Essential oil (EO) content, compositions and determination of chemotypes

**Figure 2:**
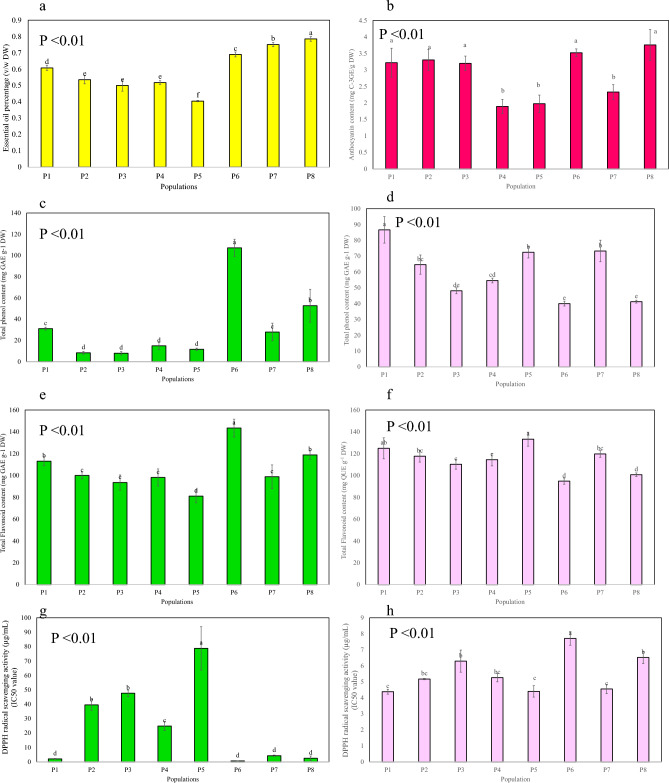
Phytochemicals and antioxidant activity in each population (**a**) essential oil content (**b**) anthocyanin content (**c**) leaves total phenol content (TPC) (**d**) flowers TPC (**e**) leaves total flavonoid contents (TFC) (**f**) flowers TFC (**g**) DPPHsc activity of leaves (**h**) DPPHsc activity of flowers.

The colors of the essential oils ranged from colorless with a mild scent to yellow, dark yellow, and greenish-yellow with a mild to very strong fragrance. The main reason for the change in color of essential oil samples could be the alteration of the existing compounds in the EOs among the evaluated populations. Color changes in EO samples collected from various populations have been reported in previous report^18^. The EO content varied among populations, ranging from 0.40 to 0.78 (V/W) with the lowest and highest amounts was obtained in populations P5 and P8, respectively (Fig. 2a). The highest EO contents were found in the region with the lowest elevation and a westward slope which received ample sunlight throughout the day and predominantly featured dry slopes. In this plant, a previous report has stated that the EO content was 0.4 v/w, which is in line with the lowest reported level of EO in the current study^14^.

**Figure 3:**
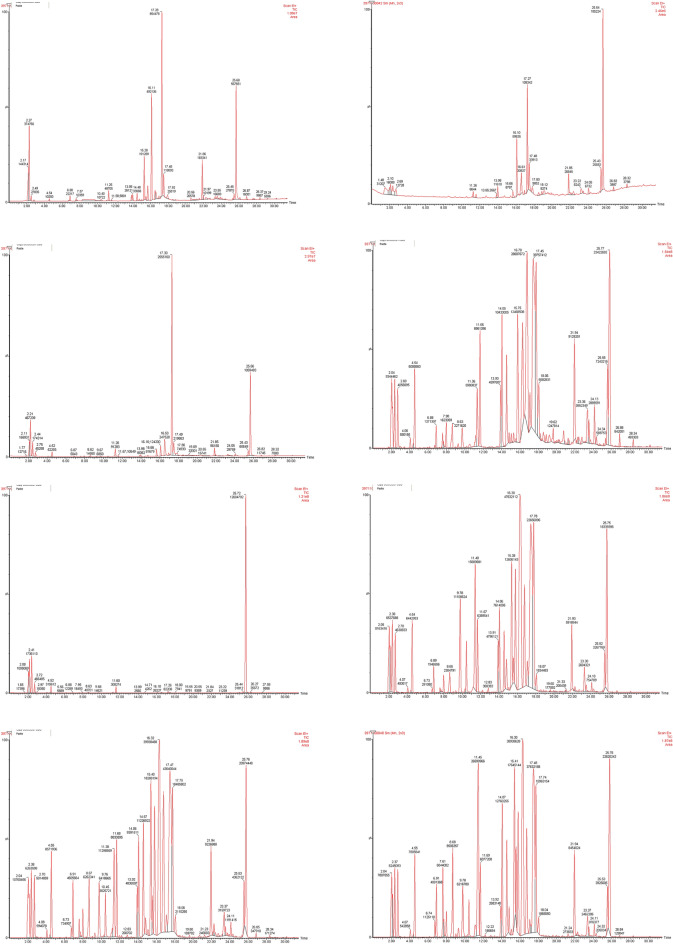
GC/MS chromatograms of eight samples from collected population.

According to the GC and GC–MS analysis, 26 compositions were identified, which constituted approximately 99–99.50% of the EOs among the populations (Table 3). The main chemical groups found in EOs of *H. longiflorus* included hydrocarbon monoterpenes (0.17–4.70), oxygenated monoterpenes (0.64–15.82), sesquiterpene hydrocarbons (0.03–22.56), oxygenated sesquiterpenes (1.12–68.43), phthalates (9.73–94.72), and other compound groups (0.94–7.39). The main predominant compounds in the population's EO include tau-cadinol (0.62–55.56), mono (2-ethylhexyl) phthalate (8.10–94.70), elemol (0.21–19.11), β- spathulenol (0.08–14.39), 4-terpineol (0.23–10.19) and β-eudesmol (0.21–9.94). The highest percentage of tau-cadinol was recorded in population P3, while mono (2-ethylhexyl) phthalate was predominant in population P5. Elemol was found to be the most prevalent in population P6, β-spathulenol in population P4, 4-terpineol in population P8, and β-eudesmol in population P4. The diversity of predominant compounds (tau-cadinol and mono (2-ethylhexyl) phthalate) and their percentage in the collected samples was highly variable due to the fact that the samples were gathered from different regions. This characteristic represents a positive aspect of plants collected from natural habitats, as valuable chemotypes for domestication and breeding purposes are identified. The high diversity of essential oil compounds has also been reported in other studies conducted on different plant species. The composition and quantity of essential oils in plants are influenced by various factors including sexual, seasonal, ontogenetic, genetic variations, as well as ecological and environmental characteristics^18,19^. Chromatograms of GC/MS for eight population are illustrated in Fig. 3.

**Table 3:** Chemical composition of essential oils of *Hymenocrater longiflorus* Benth. samples collected from eight natural habitats.

	Names	Formula	RT	RI	P1 (G1-25)	P2 (G26-50)	P3 (G51-75)	P4 (G76-100)	P5 (G101-125)	P6 (G126-150)	P7 (G151-175)	P8 (G176-200)
No	Chemotype classification (HCA)				I	I	III	III	II	III	III	III
1	α-Thujene	C_10_H_16_	6.73	928	0.05	0.01	0.00	0.03	0.00	0.12	0.28	0.43
2	α-Pinene	C_10_H_16_	6.88	935	0.81	0.30	0.11	0.69	0.09	0.78	1.78	1.56
3	β-Pinene	C_10_H_16_	7.56	980	0.38	0.02	0.03	0.41	0.09	0.04	0.28	2.31
4	Decane	C_10_H_22_	7.98	1002	0.02	0.06	0.01	0.82	0.14	0.50	0.61	0.49
5	Eucalyptol	C_10_H_18_O	8.63	1033	0.36	0.16	0.32	1.14	0.31	0.96	2.42	3.25
6	(Z)-β-Terpinolene	C_10_H_16_	9.01	1067	0.02	0.07	0.01	0.00	0.00	0.01	0.02	0.06
7	( +)-3-Carene	C_10_H_16_	9.34	1083	0.04	0.17	0.02	0.05	0.00	0.06	0.13	0.34
8	Linalool	C_10_H_18_O	9.76	1097	0.11	0.05	0.15	0.94	0.11	4.46	2.48	2.37
9	Cyclohexen,3,4 di ethenyl-3-methyl	C_11_H_16_	10.45	1129	0.39	0.30	0.05	0.13	0.06	1.14	1.48	1.18
10	4-Terpineol	C_10_H_18_O	11.25	1167	**1.77**	**1.31**	**1.99**	**3.01**	**0.23**	**6.37**	**4.36**	**10.19**
11	2-Propenoic acid 2-ethyl hexyl ester	C_19_H_28_O_2_	11.58	1192	0.21	0.66	0.23	4.51	2.32	2.56	3.41	2.32
12	α-Copaene	C_15_H_24_	13.85	1375	0.84	0.70	0.17	2.31	0.01	1.92	1.87	1.02
13	β-Bourbonene	C_15_H_24_	13.98	1384	0.95	2.29	0.23	5.25	0.02	3.05	3.63	4.87
14	trans-β-Caryophyllene	C_15_H_24_	14.48	1416	1.22	0.28	0.07	4.07	0.00	2.05	4.33	3.27
15	(Z)-β-Farnesene	C_15_H_24_	14.79	1430	0.25	0.39	0.02	0.26	0.00	1.86	0.46	0.63
16	α-Neoclovene	C_15_H_24_	15.09	1442	0.18	0.19	0.01	0.26	0.00	1.08	0.23	0.21
17	γ-Cadinene	C_15_H_24_	15.28	1447	6.95	0.59	0.00	0.28	0.00	5.06	7.06	6.74
18	β-Cadinene	C_15_H_24_	15.58	1458	0.44	0.57	0.00	0.13	0.00	0.52	0.70	1.24
19	γ-Muurolene	C_15_H_24_	15.8	1472	2.21	1.73	1.78	6.27	0.00	3.85	4.28	3.99
20	Elemol	C_15_H_26_O	16.3	1537	**17.89**	**11.77**	**2.70**	**6.33**	**0.21**	**19.11**	**15.42**	**14.87**
21	β-Spathulenol	C_15_H_24_O	16.74	1582	**0.82**	**6.10**	**5.38**	**14.39**	**0.08**	**4.99**	**6.96**	**4.37**
22	Tau. Cadinol	C_15_H_26_O	17.28	1639	**31.07**	**21.39**	**55.56**	**19.99**	**0.62**	**18.71**	**16.85**	**14.37**
23	β-Eudesmol	C_15_H_26_O	17.48	1654	**4.29**	**6.69**	**4.78**	**9.94**	**0.21**	**9.09**	**7.14**	**6.10**
24	Isobutyl phthalate	C_16_H_22_O_4_	21.86	1841	6.67	5.30	2.09	4.59	0.02	2.37	3.56	3.23
25	1-Monopalmitin	C_19_H_38_O_4_	23.37	1915	0.61	1.63	0.65	1.94	0.08	1.08	1.22	0.94
26	Mono(2-ethylhexyl) phthalate	C_16_H_22_O_4_	25.64	2023	**20.64**	**36.56**	**23.04**	**11.78**	**94.70**	**7.36**	**8.10**	**8.64**
	Total identifed	–	–	–	99.20	99.3	99.4	99.5	99.3	99.1	99.05	99
	Monoterpene Hydrocarbons	–	–	–	1.31	0.56	0.17	1.19	0.18	1.01	2.49	4.70
	Oxygenated monoterpenes	–	–	–	2.24	1.51	2.45	5.09	0.64	11.79	9.26	15.82
	Sesquiterpene hydrocarbons	–	–	–	13.05	6.76	2.29	18.82	0.03	19.40	22.56	21.98
	Oxygenated Sesquiterpenes	–	–	–	54.07	45.96	68.43	50.64	1.12	51.90	46.36	39.71
	Phthalates	–	–	–	27.30	41.86	25.13	16.37	94.72	9.73	11.66	11.87
	Others	–	–	–	1.23	2.65	0.94	7.39	2.60	5.28	6.72	4.93

Major constituents are in [bold].

Previous studies have primarily focused on analyzing the chemical composition with only a single population that have shown that hedycaryol, α-cadinol, β-bourbonnene, α-pinene, β-caryophyllene, β-ocimene, **γ**-cadinol, *p*-menth-1-en-8-ol, 1,8-cineole, carvone, thymol, estragole, and β-citronellol were the major constituents of the plant^12,13,15,16^. The oxygenated sesquiterpene, tau-cadinol, have also been identified in higher proportions within the EO of *Pulicaria crispa* (53.5%) and *Pulicaria arabica* (38.6%) that have been found to possess insecticidal activity^20^. Furthermore, tau-cadinol, which serves as a major constituent of *Ipomoea carnea* (38.6%), has been recognized for its significant antibacterial properties^21^. *Eupatorium odoratum* EO, with the predominant compound tau-Cadinol (20.10%), has shown antimicrobial and antioxidant effects^22^. α-cadinol as isomeric form of tau-cadinol with (20.43%) was the second dominant component of *H. longiflorus*^16^. Mono (2-ethylhexyl) phthalate, in addition to being a plasticizer, possesses various biological properties including cytotoxicity, antioxidant, anti-inflammatory, antimicrobial and antiviral activity^23^. In this regard, chemotypes with only a few dominant constitute are the attention of different industries^24^. Research has demonstrated that certain types of algae have the ability to produce phthalates such as di-*n*-butyl phthalate and mono (2-ethylhexyl) phthalate as a defense mechanism against environmental stressors. These phthalates can be released into the surrounding environment, potentially impacting the delicate balance of aquatic ecosystems. Within algal cells, these synthesized phthalates are believed to be stored in cell membranes to help maintain the flexibility and resilience of the cells. These findings indicate that phthalate production may be a widespread occurrence, both on land and in marine environments^23^. Mono (2-ethylhexyl) phthalate has been reported as a predominant compound in all parts of the plants *Cirsium japonicum* at 16%, *Salvinia natans* at 29.3%, and *Eichhornia crassipes*, as well as in the flowers of *Osmanthus fragrans* at 26.5%, serving as a natural component. These compounds have shown beneficial effects such as reducing soil-borne diseases, improving soil quality, and promoting plant growth^23^. The process of selecting new plant crops for the pharmaceutical industry is market-driven and requires careful consideration of various factors. This includes identifying valuable natural bioactive compounds, conducting extensive extraction procedures, and developing products that meet market demands. It is a complex and time-consuming process that requires expertise and careful evaluation^25^. In the present study, P3 and P5 with dominant compounds tau-cadinol and mono (2-ethylhexyl) phthalate can be used for domestication and extensive cultivation after supplementary studies.

**Figure 4:**
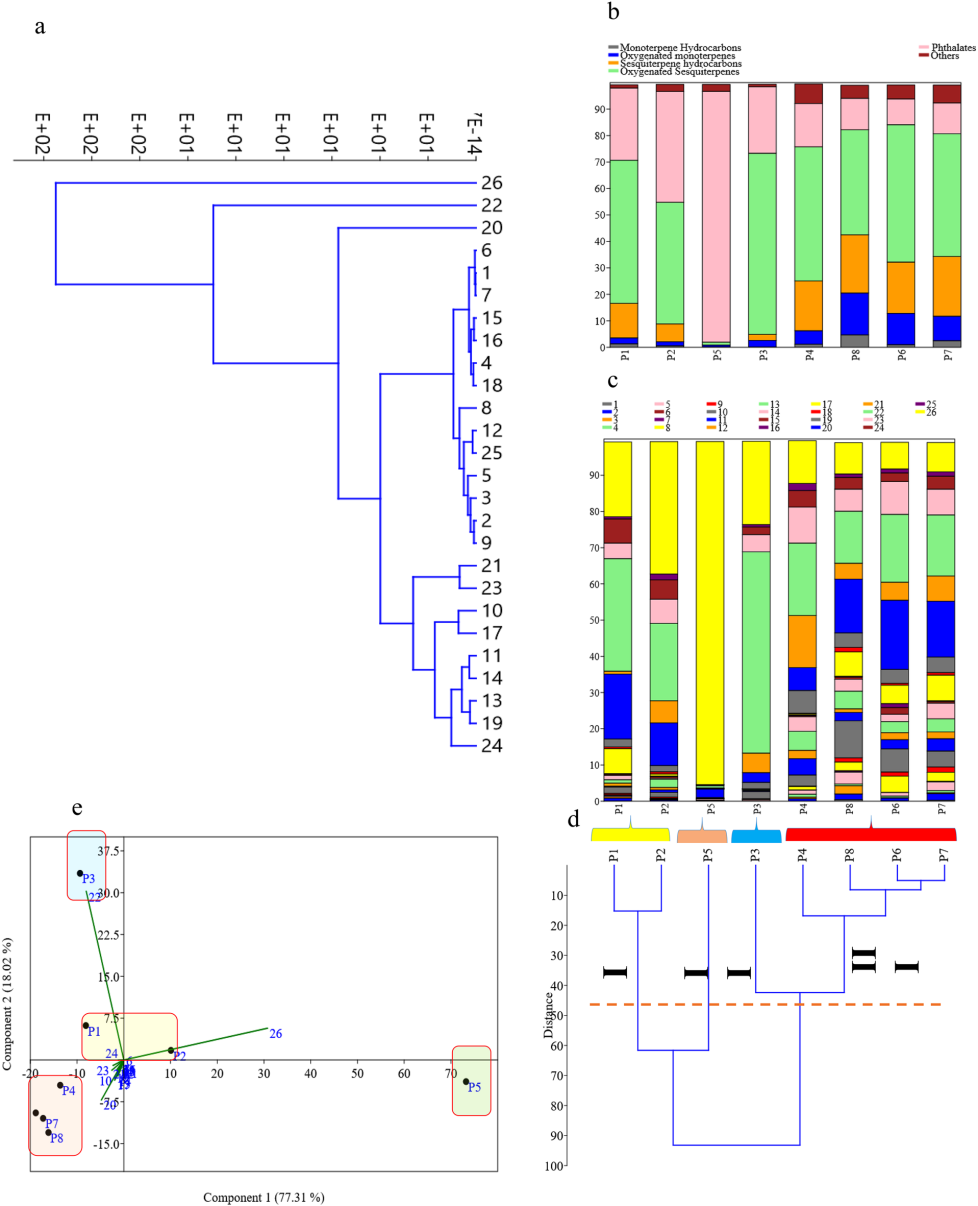
Multivariate analysis and stacked column chart of the 26 constituents among eight population (**a**) Hierarchical clustering of 26 EO constituents (**b**) stacked column of EO classification group’s percentage (**c**) stacked column of EO based on 26 EO constituents (**d**) classification and chemotyping of eight population based on EO (**e**) Di-plot of two first components based on PCA analysis of EO compositions.

In order to classify and determine the chemotype of eight populations, the EO components were subjected to hierarchical cluster analysis (HCA) in line with stacked column charts (Fig. 4a–d). The dendrograms of the eight populations, as shown in Fig. 4d, were divided into three main groups, indicating a distinct chemotypes. Additionally, a stacked column chart of the 26 constituents, as well as the EO component classification, were provided alongside the dendrogram to demonstrate which constituents correlated with the chemotypes and populations Fig. 4b,c. In chemotype I, which consisted of populations P1 and P2, three major components were identified, including mono (2-ethylhexyl) phthalate, tau-cadinol, and α-elemol. Chemotype II, which only had one population (P5), was rich in mono (2-ethylhexyl) phthalate but lacked tau-cadinol and α-elemol. Chemotype III, which included populations P3, P4, P6-8, contained tau-cadinol, β-eudesmol, and 4-terpineol. Furthermore, in the clustering of compounds, it was found that three compounds, mono(2-ethylhexyl) phthalate (26), tau-cadinol (22), and elemol (20), had the greatest impact on the separation and differentiation of populations (Fig. 4e). Additionally, the principal component analysis (PCA) yielded similar results, where these three compounds (26, 22 and 20) had the greatest impact on the clustering. Specifically, compound 26 had the highest concentration in population P5, compound 22 was predominant in P3 and to some extent in P1, and compound 20 was observed in P4 and P6-8. These two principal components (PC1 and PC2) were able to explain more than 95% of the variations in population compositions in terms of phytochemical compounds and chemotypes determination.

### Flowers anthocyanin content

The results of the analysis of variance showed that the populations of *H. longiflorus* had a significant difference in terms of total anthocyanin content (*p* < 0.01). The evaluated range of anthocyanin content in the populations varied from 1.89 to 3.75 mg cyanidin-3-glucoside equivalent C-3GE/g DW (Fig. 2b). The order of anthocyanin content, from highest to lowest, was observed in populations P1-3, P6, P8 > P4, P5, P7. However, there was no statistically significant difference between populations P1, P2, P3, P6, and P8. Although anthocyanins are most recognized as pigments contributing to coloration in fruits and flowers usually in attracting pollinators, however, high levels of these compounds can encourage the use of flowers as medicinal organs^26^.

### Total phenolic content (TPC) and total flavonoid content (TFC)

Significant differences (*p* < 0.01) were observed in the levels of TPC and TFC in leaf and flower samples obtained from eight distinct populations. The TPC in the leaf samples exhibited a wide range, spanning from 7.89 to 117.18 mg gallic acid equivalent (GAE)/g DW (Fig. 2c). Notably, the population denoted as P6 exhibited the highest TPC, followed by P8, P1, P7, and populations P2 to P5. Conversely, the flower samples displayed a TPC ranging from 39.98 to 86.62 mg GAE/g DW, with the highest content observed in population P1 and the lowest in populations P8 and P6 (Fig. 2d). Furthermore, the TFC varied from 81.04 to 143.46 mg quercetin equivalent (QUE)/g DW in leaf samples and from 94.82 to 133.26 mg QUE/g DW in flower samples across the different populations (Fig. 2e,f). The order of TFC in leaf samples from populations, from highest to lowest, was as follows: P6 > P8 > P1 > P2-4, P7 > P5. Additionally, in flower samples, the highest and lowest amounts were observed in populations P5 and P6 respectively. It has been reported that phenolic compounds have antimicrobial and antioxidant properties, helping plants prevent infections from pathogens and pathogenic microorganisms. Additionally, their presence in plant tissues protects them against the toxic effects of reactive oxygen species^27^.

### DPPH free radical scavenging activity

The antioxidant properties of leaf and flower samples from *H. longiflorus* populations were evaluated using the DPPH free radical scavenging activity method. The results showed a significant statistical difference (*p* < 0.01) in the fifty percentage inhibition concentration (IC50) values of DPPH scavenging activity (DPPHsc) among eight populations. The IC50 values ranged from 0.65 to 78.74 µg/ml in leaf samples and from 4.4 to 7.71 µg/ml in flower samples (Fig. 2g,h). To facilitate comparison of the samples with a standard antioxidant, the antioxidant power of ascorbic acid DPPHsc (IC50) was determined to be 3.24 ± 0.12 µg/ml. A lower IC50 value indicates higher antioxidant activity. Among the leaf samples, populations P6, P1, P7, and P8 exhibited the highest antioxidant potential, with lower IC50 values that were not statistically significant from each other. Similarly, among the flower samples, populations P1, P5, and P7 exhibited the highest antioxidant activity with lower IC50 values.

### Correlations and multivariate analysis of combined data

**Figure 5:**
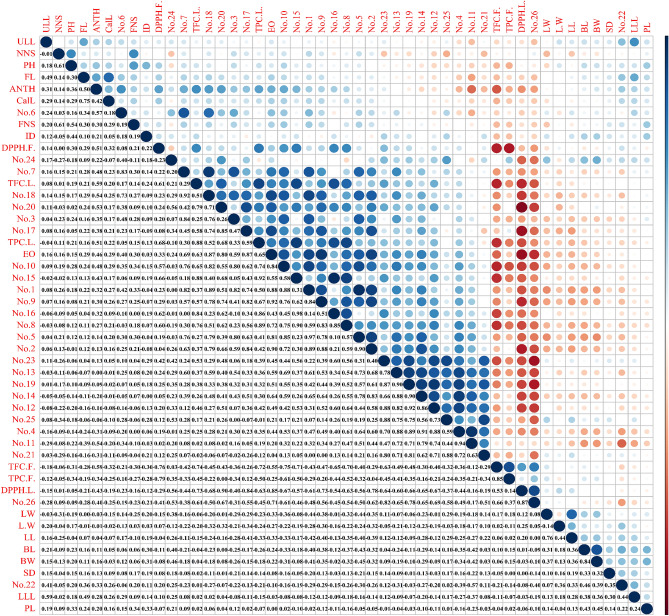
Simple correlation among 49 morphological and chemical traits obtained from 200 individuals from eight population of *Hymenocrater longiflorus.*

Correlation analysis is a valuable tool for investigating relationships between traits, and it has practical implications in plant breeding and domestication. In Fig. 5, the correlation between traits is presented both quantitatively and visually, with large blue points indicating a positive correlation and large red points indicating a negative correlation. Among the morphological traits, the length and width of the bracts (BL, BW) exhibited a strong positive correlation (0.84). Moreover, these morphological traits showed a positive correlation with compound 24 of EO (BL = 0.40, BW = 0.46), while displaying a negative correlation with compounds 1 and 5. These correlations with phytochemical traits can contribute to cost-effective analysis in the selection process of these plants and serve as valuable indicators for marker-assisted selection (MAS)^28^. The negative correlation observed between DPPH leaf (DPPH L) IC50 and TFC (-0.73), TPC (-0.63), compound 20 (-0.90), EO (-0.85), and compound 9 (-0.73) suggests that these compounds exert a significant influence on the antioxidant activity of the leaves. Additionally, DPPH flower (DPPH F) IC50 exhibited a negative correlation with TPC (-0.79) and TFC (-0.78), but a positive correlation with flower anthocyanins (0.51), compound 15 (0.66), and compound 10 (0.57). These findings underscore the substantial impact of total phenols and flavonoids on the antioxidant activity of the flower organ. Notably, a significant positive correlation was observed between components 1, 2, 5, 8, 9, 10, 14, 17, 18, 20 and EO. Furthermore, the positive correlation between leaf flavonoids (TFC L) (0.63), leaf total phenolic content (TPC L) (0.65), and the EO content indicates that conditions promoting an increase in essential oil content also lead to elevated levels of phenolic compounds. The simultaneous increase of one group of metabolites with another group is of great interest to breeders, particularly in medicinal plants where an increase in all beneficial secondary metabolites may be desired. simultaneous breeding of several attributes is interested among the breeders^29^. Additional correlations between traits are depicted in Fig. 5.

Canonical correspondence analysis (CCA) is a multivariate statistical technique used to explore the relationship between two sets of variables. In this study, CCA was employed to examine the association between phytochemical and morphological traits in 200 genotypes from 8 populations. The mean values of various morphological traits were compared with those of phytochemical components, including EO constituents and content, total anthocyanins, total phenols, and total flavonoids. Table 4 displays the results of the CCA, presenting seven canonical correlations (CC 1–7) along with their corresponding eigenvalues and the percentage of variance explained by each correlation. CC1, with an eigenvalue of 0.116, accounts for 60.13% of the total variance. CC2 and CC3 explain 16.27 and 10.83% of the variance, respectively. In this multivariate analysis, positive signs ( +) indicate positive correlations between variables, while negative signs (−) indicate negative correlations. The data more than 0.4 used for CCA for explain of associations.

**Table 4:** The results of the canonical correspondence (CC) analysis, presenting seven canonical correlations (CC 1–7) along with their corresponding eigenvalues and the percentage of variance explained by each correlation among phytochemical, morphological and studied eight populations.

Phytochemical and morphological traits	Canonical correspondence (CC)						
	1	2	3	4	5	6	7
1	− 2.16	1.15	2.07	2.00	− 8.96	9.01	− 5.85
2	− 1.46	− 0.06	2.98	1.76	− 2.67	4.85	− 4.48
3	− 1.73	0.60	0.82	1.37	− 14.76	5.91	18.40
4	− 1.08	0.11	5.22	− 3.78	− 1.23	2.15	1.04
5	− 1.45	0.30	2.88	0.09	− 6.57	7.44	− 2.88
6	− 0.62	− 0.79	− 1.39	2.82	− 8.21	− 8.47	− 9.58
7	− 1.27	− 0.04	0.48	1.25	− 10.05	− 1.48	− 4.54
8	− 2.31	2.88	1.38	− 2.56	0.43	3.85	− 8.96
9	− 1.85	1.40	1.83	1.48	− 1.97	4.95	− 13.03
10	− 1.71	0.94	0.44	− 1.09	− 4.93	3.55	1.40
11	− 0.21	0.60	4.83	− 3.16	− 0.69	2.18	0.52
12	− 1.29	− 0.44	3.91	− 2.20	1.50	− 1.18	− 0.47
13	− 1.24	− 0.55	3.62	− 2.32	− 3.45	− 1.64	1.39
14	− 1.47	− 0.75	5.16	− 1.31	− 1.70	3.73	0.11
15	− 2.19	3.18	− 0.41	− 2.42	2.39	− 3.44	− 6.28
16	− 2.10	2.92	0.20	− 3.20	4.70	− 4.52	− 3.71
17	− 1.93	0.85	1.27	4.59	− 0.45	4.89	− 1.22
18	− 1.56	0.61	0.70	2.39	− 6.08	− 0.42	− 4.65
19	− 1.10	− 1.13	3.08	− 2.35	− 0.36	0.29	2.88
20	− 1.38	0.32	0.63	1.98	0.62	− 1.87	− 2.32
21	− 0.48	− 2.42	3.48	− 4.57	− 0.30	− 2.34	− 0.19
22	− 0.19	− 2.94	− 2.15	− 0.85	2.22	2.38	0.69
23	− 0.89	− 0.97	1.62	− 2.14	0.43	− 2.28	− 1.43
24	− 0.59	− 1.91	1.22	1.74	0.64	− 4.88	3.42
25	− 0.55	− 1.57	2.35	− 2.09	− 0.58	− 4.93	− 2.70
26	2.98	2.23	0.04	0.83	− 0.06	− 0.13	1.02
EO	− 0.40	− 0.09	0.69	0.21	− 0.63	1.17	− 1.47
TPC leaf (L)	− 1.85	3.02	− 0.55	− 1.21	1.68	0.46	0.60
TPC fruit (F)	0.44	− 0.37	1.14	1.46	1.07	0.14	− 0.15
TFC (L)	− 0.28	0.11	0.26	− 0.29	0.27	− 0.18	0.56
TFC (F)	0.37	− 0.26	0.78	0.19	0.27	0.42	0.11
DPPH (L)	3.15	0.22	− 0.60	− 2.32	− 0.32	0.40	− 1.42
DPPH (F)	− 0.19	0.05	− 0.17	− 1.22	− 0.16	0.41	0.01
Anthocyanin	− 0.24	− 0.15	− 0.71	0.17	− 0.61	− 0.36	− 0.17
P1	− 0.11	− 0.07	− 0.03	0.22	0.09	− 0.01	0.04
P2	0.18	− 0.09	− 0.05	0.03	− 0.05	− 0.11	− 0.03
P3	0.18	− 0.25	− 0.23	− 0.10	0.03	0.06	− 0.01
P4	0.01	− 0.18	0.27	− 0.19	0.02	− 0.03	0.04
P5	0.76	0.27	0.04	0.01	0.00	0.03	0.01
P6	− 0.41	0.26	− 0.08	− 0.10	0.09	− 0.02	− 0.01
P7	− 0.18	− 0.06	0.21	0.09	0.02	0.07	− 0.05
P8	− 0.30	0.07	− 0.04	0.02	− 0.18	0.03	0.02
PH	0.21	0.11	− 0.69	− 0.03	− 0.56	0.40	0.10
NNS	− 0.42	0.39	− 0.47	0.14	− 0.25	0.61	− 0.15
FNS	0.05	− 0.12	− 0.37	0.08	− 0.65	0.62	− 0.18
IL	0.58	− 0.17	− 0.03	− 0.19	− 0.50	− 0.42	0.44
BL	0.29	− 0.65	− 0.33	0.20	0.30	− 0.38	0.27
BW	0.39	− 0.52	− 0.26	0.26	0.24	− 0.52	0.27
SD	0.54	− 0.68	− 0.23	0.04	0.06	− 0.18	− 0.28
PL	− 0.02	− 0.55	− 0.47	0.28	− 0.26	− 0.19	0.50
FL	0.30	− 0.23	− 0.55	0.44	− 0.48	0.31	− 0.23
ULL	0.25	− 0.63	− 0.39	0.30	− 0.36	0.23	− 0.24
LLL	0.34	− 0.69	− 0.45	0.15	− 0.22	− 0.18	− 0.21
CL	0.28	− 0.05	− 0.45	0.27	− 0.51	0.61	− 0.24
LL	0.35	− 0.61	− 0.25	− 0.20	0.06	− 0.62	− 0.07
LW	0.27	− 0.29	0.02	0.02	0.22	− 0.88	0.17
Eigenvalue	0.11662	0.03156	0.020996	0.013674	0.0071304	0.0030172	0.00093082
Variance (%)	60.13	16.27	10.83	7.051	3.677	1.556	0.48

In CC1, a positive association is observed between the combination of DPPH (L) and compound 26 with population 5, as well as the morphological traits stem diameter (SD) and internode distance (ID). Additionally, compound 20, along with other essential oil compounds (1–10 and 12–25) and TPC L in populations P6 and the node number per stem (NNS) trait, display positive correlations. Thus, high NNS values in populations may be linked to increase of these phytochemical traits. CC2 reveals a positive correlation between compound 1, 8, 9, 15, 16, and 26, as well as leaf total phenolic contents (TPC L) in populations P5 and P6, with the morphological traits PH and NNS. Conversely, compound 19, 21, and 22 in population P3 exhibit negative correlations with the morphological traits BL, BW, SD, PL, UUL, LLL, and LL.

**Figure 6:**
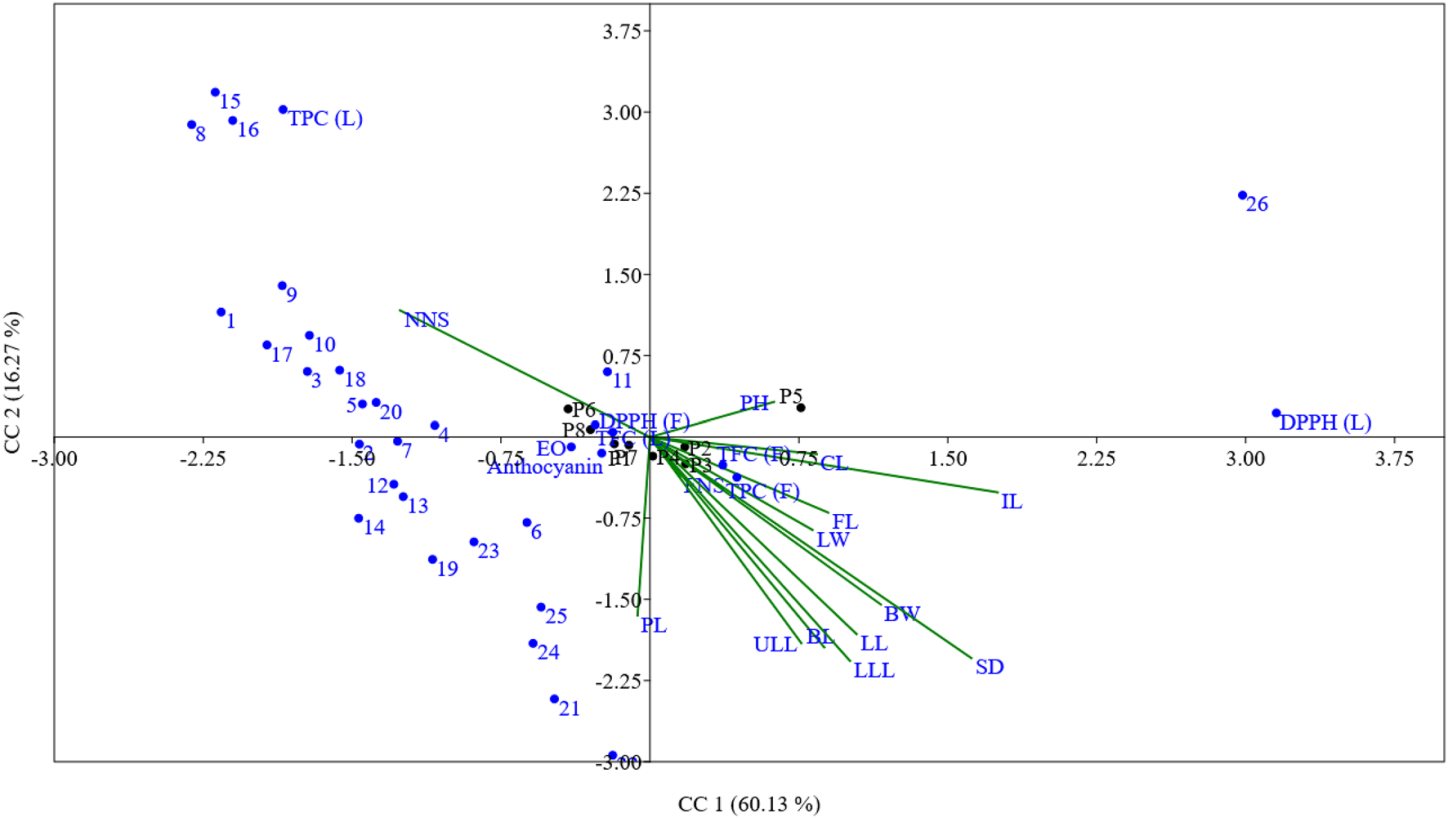
Canonical correspondence analysis of between morphological and phytochemical traits of *Hymenocrater longiflorus* populations.

Considering that CC1 and CC2 collectively explain 76.40% of the relationship between morphological and phytochemical traits, scatter plots were generated using these two canonical correlations (Fig. 6). The direction of the lines associated with phytochemical traits indicates their positive or negative correlation with other studied traits. The positive correlation between NNS and TPC (L), as well as compounds 8, 5, and 16, suggests a relationship with this morphological trait. Moreover, plant height (PH) shows a positive correlation with population P5 and the combination of essential oil 26 and DPPH (L). Conversely, most morphological traits exhibit an inverse correlation with essential oil compounds and total leaf phenols (TPC L), indicating that smaller plant stature is associated with higher levels of EO in populations.

**Figure 7:**
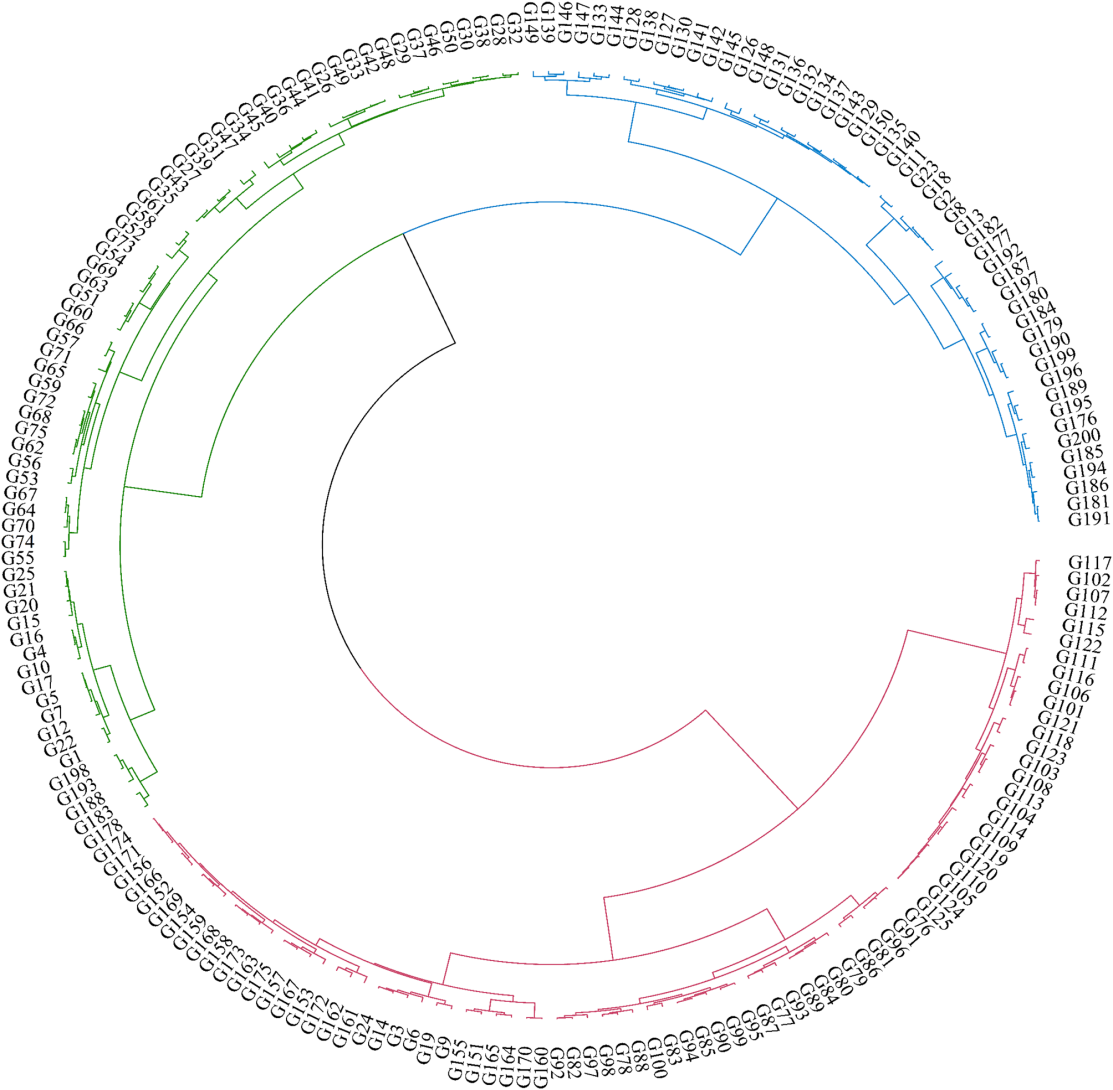
Hierarchical clustering of all combined traits among 200 individuals of *Hymenocrater longiflorus.*

To further explore the diversity of phytochemical and morphological traits among populations, cluster analysis using R software (circular cluster) was conducted (Fig. 7). The analysis divided the genotypes of all individuals in the eight studied populations into three main categories based on all the studied traits. This clustering indicates a significant variation in phytochemical and morphological traits among populations, surpassing the within-population variation. Therefore, the interbreeding between individuals from two distant populations as opposed to the interbreeding of individuals within populations can potentially lead to the emergence of new genotypes that are adaptable to different regions and contribute to the development of more effective plant breeding and cultivation.

The analysis of plant populations showed that the plant is a rich source of compounds tau-cadinol, and mono (2-ethylhexyl) phthalate. According to the EO groups, oxygenated sesquiterpenes played a major role in forming the majority of the EOs in studied populations. The role of oxygenated sesquiterpenes has been identified in insecticidal, antioxidant, and allelopathic activities, indicating that chemotypes containing these compounds could be new candidates for using industrially for this aims^7,30^. Furthermore, the study on populations demonstrated that the antioxidant activity of the flower and leaf extracts of the plant is high, suggesting their potential use as antioxidant compounds^31^. Examination of the plant extracts revealed that they are rich sources of flavonoids, phenolics, and flowers are particularly rich in anthocyanins. Therefore, the studied populations showed that the plant holds promise for domestication and improvement for widespread cultivation. The presence of mono (2-ethylhexyl) phthalate in the P5 population with a high percentage of over 94% can be a valuable source for this compound, as it possesses various biological properties, including cytotoxicity, antioxidant, anti-inflammatory, antimicrobial, and antiviral activities^23^. Three valuable chemotypes identified in the present study are as follows:

(I) mono (2-ethylhexyl) phthalate, tau-cadinol, and α-elemol; (P1 and P2).

(II) mono (2-ethylhexyl) phthalate; (P5).

(III) tau-cadinol, β-eudesmol, and 4-terpineol; (P3, P4, P6, and P8).

CCA and correlation plot analysis indicated a strong relationship between morphological and EO gradients. These properties could eliminate the need for expensive and time-consuming analytical quantification methods when selecting plants for domestication and breeding. In conclusion, the application of CCA, scatter plots, and cluster analysis provides valuable insights into the relationship between phytochemical and morphological traits in the studied populations. These findings contribute to our understanding of the diversity and interplay of these traits in the plant species under investigation.

## Methods

### Plant material

To obtain the plant material, the aerial parts of *H. longiflorus* were collected in full bloom in June from different parts of the Zagros Mountains, West of Iran. Sampling was done for academic purposes, with the permission of the University and in accordance with relevant institutional, national, and international guidelines and legislation. Sampling was in accordance with the IUCN guideline on research on endangered species and the Convention on the Trade in Endangered Species of Wild Fauna and Flora. Twenty five individual samples were randomly selected in each population from eight collection site. The species were identified by Mr. Hossein Maroofi, from Research Center of Agriculture and Natural Resources of Kurdistan, Sanandaj, Iran. Then was labeled with a voucher specimen (No. 1521) in the herbarium of the Department of Horticultural Science, Urmia University, Iran. The aerial parts were dried in the shade at room temperature for two weeks. To identify the habitats of these plants, local people were interviewed, previous articles and books were consulted (Table 1).

### Essential oil isolation and GC/MS analysis

The EO content was determined by extracting 35 g of dried plant materials using the water distillation method with a Clevenger-type apparatus over a 3 h period and the EO content expressed in % (v/w). The obtained oils were then stored in securely sealed vials with foil covers at 4 °C prior to analysis. Gas chromatography-mass spectrometry (GC/MS) analyses were conducted using a Thermo Finnigan capillary gas chromatograph directly linked to the mass spectrometer system (model GC TRACE; TRACE MS plus). A non-polar fused silica capillary column (HP-5MS, 30 m × 0.250 mm, 0.25 μm film thickness) was utilized. The injector temperature was set at 280 °C. The GC oven temperature was programmed as follows: initially, the oven temperature was set at 40 °C for 2 min, then increased to 160 °C at a rate of 3 °C/min, and finally raised at a rate of 5 °C/min to 280 °C, where it was held for 2 min. Helium used as the carrier gas at a flow rate of 1 ml/min, and the ionization energy was set at 70 eV. Without any dilution, 1 μL of each essential oil sample was manually injected into the GC, and the split ratio was 1:60^18^. The injection conditions for both GC and GC–MS were identical. The percentages of chemical constituents were determined using the area normalization method, without considering response factors. Retention indices (RI) were calculated using the retention times of injected *n*-alkenes (C6–C24) under the same experimental conditions. Compound identification was achieved by comparing the RIs with those available in NIST and other relevant literature sources^32,33^, and the mass spectra of the compounds were identified using the installed libraries on X-Calibur (2.07) software.

### Preparation of methanolic extracts

The leaf and flower samples from each region were first dried and finely powdered, with 1 g of each sample being used for extraction. Following this, 15 ml of 80% methanol extract was added to each gram of sample. The samples were then subjected to extraction in an ultrasonic bath for 30 min at 25 °C and 120 Hz waves using an Elmasonic E 120 Hz apparatus from Elma Schmidbauer GmbH, Germany. The resulting extracts were filtered with whatman filter paper before being stored at 4 °C.

### Determination of total phenolic and flavonoid content

The total phenolic content (TPC) was determined using the Foline-Ciocalteu procedure, following the method described previously with some modifications^34^. Specifically, 10 µl of each extract were combined with approximately 1200 µl of Foline-Ciocalteu (10%), followed by the addition of 960 µl of sodium carbonate (7%) and 180 µl of distilled water. The solution was thoroughly mixed and then incubated in darkness for 30 min. Subsequently, the absorbance was measured at 760 nm using a spectrophotometer (Dynamica HALO DB-20, UK). For the determination of total flavonoids, the aluminum chloride colorimetric method was employed with slight adjustments^27^. Initially, 15 µl of the extract was mixed with 150 µl of NaNO_2_ (5%) and left at room temperature for 15 min. Then, 300 µl of AlCl_3_ (10%) and 1 ml of NaOH were added to the solution. After a 30 min. incubation period, the mixture exhibited a yellow color, and the absorbance was measured at 380 nm.

### DPPH free radical scavenging assay

The radical scavenging activity of the extracts was determined using the DPPH assay, with slight modifications to the procedure reported by Yahia and coworkers^35^. Three different concentrations (2, 5, and 10 ppm) of methanol extracts from each region were mixed with 2 ml of 0.1 mM methanol DPPH solution. The mixture was gently shaken and left at room temperature for 15 min. The absorbance of the samples was measured at 517 nm using a UV–visible spectrophotometer, with methanol (80%) used as the blank and DPPH solution without extract used as the control. Ascorbic acid solution was used as the standard, with absorbance determined in the same way as the plant extracts.

The formula used to calculate the inhibition percentage is as follows:

$$ {\text{Inhibition}}\% \, = \frac{{A_{B} - A_{A} }}{{A_{B} }} \times 100 $$

Here, $A_{A}$ represents the absorbance of the DPPH solution with extract, and $A_{B}$ represents the absorbance of the DPPH solution without extract, which serves as the control.

### Anthocyanin determination

Anthocyanins, which are a type of phenolic compound, were analyzed using the previous established method^36^. Dried flower samples (0.1 g) were ground into a fine powder and then mixed with 1.5 mL of acidic methanol (a solution of methanol and hydrochloric acid in a 1:99 ratio). The resulting mixture was centrifuged to separate the components. To measure the total anthocyanin content (TAC), the pH difference procedure was employed. This involved preparing two buffers with different pH values (pH = 1 and pH = 4.5). Subsequently, 2.5 mL of the pH 1 buffer was added to the extract, followed by the addition of 2.5 mL of the pH 4.5 buffer to 100 ppm of the extract. The absorbance of the resulting solution was measured at 520 nm and 700 nm using a spectrophotometer.

$$ {\text{TAC }}\left( {{\text{mg}}/100{\text{g}}} \right) = \frac{A \times MW \times DF \times 1000}{{\varepsilon \times C}} $$

## Statistical analysis

The eight populations of *H. longiflorus* were classified and grouped based on ward distances by analyzing the essential oil composition data matrix using hierarchical cluster analysis (HCA) and principal component analysis (PCA) with PAST software (version 4.03). Additionally, canonical corresponding analysis (CCA) was conducted on the morphological, phytochemicals, and essential oil content and composition using the same software. Heat-map cluster and correlation analysis for combined data of all genotypes were obtained using RStudio (version 1.2.5019) URL http://www.rstudio.com/. Furthermore, analysis of variance with completely randomized design (CRD) (ANOVA) and means comparisons with Duncan's Multiple Range test (DMRT) were carried out using SAS software version 9.4.

### Ethical approval

Plant sampling were comply with the IUCN Policy Statement on Research Involving Species at Risk of Extinction and the Convention on the Trade in Endangered Species of Wild Fauna and Flora.

## Data Availability

The datasets generated during and/or analysed during the current study are available from the corresponding author on reasonable request.

## References

[1] Rahman MM (2022). Natural therapeutics and nutraceuticals for lung diseases: traditional significance, phytochemistry, and pharmacology. Biomed. Pharmacother..

[2] Shahgholian N (2022). Introduction to nutraceuticals and natural products. Handb. Nutraceuticals Nat. Prod. Biol. Med. Nutr. Prop. Appl..

[3] Çelebi Ö (2023). Chemical composition, biological activities, and surface tension properties of Melissa officinalis L. essential oil. Turk. J. Agric. For..

[4] Tomar O (2023). Determination of some quality properties and antimicrobial activities of kombucha tea prepared with different berries. Turk. J. Agric. For..

[5] Chakrabartty I (2022). Exploration of Lamiaceae in cardio vascular diseases and functional foods: Medicine as food and food as medicine. Front. Pharmacol..

[6] Mseddi K (2020). as a promising source of potent bioactive compounds with its pharmacological properties: *In vitro* and *in silico* analysis. Arab. J. Chem..

[7] Fattahi B, Nazeri V, Kalantari S, Bonfill M, Fattahi M (2016). Essential oil variation in wild-growing populations of Salvia reuterana Boiss. collected from Iran: Using GC–MS and multivariate analysis. Ind. Crops Prod..

[8] Tohidi B, Rahimmalek M, Arzani A (2017). Essential oil composition, total phenolic, flavonoid contents, and antioxidant activity of Thymus species collected from different regions of Iran. Food Chem..

[9] Jamzad, Z. Lamiaceae in Flora of Iran. Vol. 76 24–626 (Research Institute of Forests & Rangelands, 2012).

[10] Al-Anee RS (2015). Chemical characterization, antioxidant and cytotoxic activities of the methanolic extract of *Hymenocrater longiflorus* grown in Iraq. Z. Naturforsch. C.

[11] Morteza-Semnani K, Ahadi H, Hashemi Z (2016). The genus *Hymenocrater*: a comprehensive review. Pharm. Biol..

[12] Taherpour AA (2011). Chemical compositions of the essential oil and calculation the biophysicochemical coefficients of the components of *Hymenocrater longiflorus* Benth. Iran. Nat. Sci..

[13] Saed-Moucheshi A, Mozafari AA (2022). Alternate gene expression profiling of monoterpenes in *Hymenocrater longiflorus* as a novel pharmaceutical plant under water deficit. Sci. Rep..

[14] Ahmadi F, Sadeghi S, Modarresi M, Abiri R, Mikaeli A (2010). Chemical composition, *in vitro* anti-microbial, antifungal and antioxidant activities of the essential oil and methanolic extract of *Hymenocrater longiflorus* Benth., of Iran. Food Chem. Toxicol..

[15] Taran M, Karimi N, Abdi J, Sohailikhah Z, Asadi N (2013). Larvicidal effects of essential oil and methanolic extract of *Hymenocarter longiflorus* (Lamiaceae) against *Echinococcus granulosus*. J. Essent. Oil Bear. Pl..

[16] Shahriari S, Khanahmadi M, Tahvilian R (2013). The study of essential oil of *Hymenocrater longiflorus* Benth growing in Paveh. J. Rep. Pharm. Sci..

[17] Mao R, He Z (2020). Pinellia ternata (Thunb.) Breit: A review of its germplasm resources, genetic diversity and active components. J. Ethnopharmacol..

[18] Salimi F, Fattahi M, Hamzei J (2022). Phenolic contents, composition and antioxidant activity of essential oils obtained from Iranian populations of *Apium graveolens*, and their canonical correlation with environmental factors. Biochem. Syst. Ecol..

[19] Modareskia M, Fattahi M, Mirjalili MH (2022). Thymol screening, phenolic contents, antioxidant and antibacterial activities of Iranian populations of Trachyspermum ammi (L.) Sprague (Apiaceae). Sci. Rep..

[20] Yusufoglu HS (2021). Chemical composition of essential oils of *Pulicaria* species growing in Saudi Arabia and activity for Mediterranean fruit fly, *ceratitis capitata*. Phytochem. Lett..

[21] Abd-ElGawad AM (2022). Essential oil of *Ipomoea carnea*: chemical profile, chemometric analysis, free radical scavenging, and antibacterial activities. Sustainability.

[22] Choudhary, E., Bithel, N., Sharma, T., Saini, P. & Rajput, M. GC-MS Characterization of *Eupatorium odoratum* (L.) Leaves Essential Oil and Evaluation of *In vitro* Antimicrobial and Antioxidant Activity. J. Pure Appl. Microbiol. 17 (2023)

[23] Huang L (2021). Phthalic acid esters: Natural sources and biological activities. Toxins.

[24] Jalaei Z, Fattahi M, Aramideh S (2015). Allelopathic and insecticidal activities of essential oil of *Dracocephalum kotschyi* Boiss. from Iran: A new chemotype with highest limonene-10-al and limonene. Ind. Crop. Prod..

[25] Patra JK, Das G, Lee S, Kang S-S, Shin H-S (2018). Selected commercial plants: A review of extraction and isolation of bioactive compounds and their pharmacological market value. Trends Food Sci. Technol..

[26] Pervaiz T, Songtao J, Faghihi F, Haider MS, Fang J (2017). Naturally occurring anthocyanin, structure, functions and biosynthetic pathway in fruit plants. J. Plant Biochem. Physiol.

[27] Kumar S, Abedin MM, Singh AK, Das S (2020). Role of phenolic compounds in plant-defensive mechanisms. Plant Phenol. Sustain. Agric..

[28] De Mori G, Cipriani G (2023). Marker-assisted selection in breeding for fruit trait improvement: A Review. Int. J. Mol. Sci..

[29] Marulanda JJ (2021). Optimum breeding strategies using genomic and phenotypic selection for the simultaneous improvement of two traits. Theor. Appl. Genet..

[30] Abd-ElGawad AM, Elshamy AI, El-Nasser El Gendy A, Al-Rowaily SL, Assaeed AM (2019). Preponderance of oxygenated sesquiterpenes and diterpenes in the volatile oil constituents of Lactuca serriola L. revealed antioxidant and allelopathic activity. Chem. Biodivers..

[31] Bhavaniramya S, Vishnupriya S, Al-Aboody MS, Vijayakumar R, Baskaran D (2019). Role of essential oils in food safety: Antimicrobial and antioxidant applications. Grain Oil Sci. Technol..

[32] Adams RP (2007). Identification of essential oil components by gas chromatography/mass spectrometry.

[33] Davies N (1990). Gas chromatographic retention indices of monoterpenes and sesquiterpenes on methyl silicon and Carbowax 20M phases. J. Chromatogr. A.

[34] Ebrahimzadeh, M. A., Pourmorad, F. & Bekhradnia, A. R. Iron chelating activity, phenol and flavonoid content of some medicinal plants from Iran. Afr. J. biotechnol. 7 (2008)

[35] Yahia Y (2019). Phenolic profile, antioxidant capacity and antimicrobial activity of Calligonum arich L, desert endemic plant in Tunisia. S. Afr. J. Bot..

[36] Alizadeh Z, Fattahi M (2021). Essential oil, total phenolic, flavonoids, anthocyanins, carotenoids and antioxidant activity of cultivated Damask Rose (*Rosa damascena*) from Iran: With chemotyping approach concerning morphology and composition. Sci. Hortic..

